# Imitation versus serendipity in ranking dynamics

**DOI:** 10.1098/rsos.240177

**Published:** 2024-07-24

**Authors:** Federica De Domenico, Fabio Caccioli, Giacomo Livan, Guido Montagna, Oreste Nicrosini

**Affiliations:** ^1^ Dipartimento di Fisica, Università degli Studi di Pavia, Via A. Bassi 6, 27100 Pavia, Italy; ^2^ Istituto Nazionale di Fisica Nucleare, Sezione di Pavia, Via A. Bassi 6, 27100 Pavia, Italy; ^3^ Department of Computer Science, University College London, 66–72 Gower Street, London WC1E 6EA, UK; ^4^ Systemic Risk Centre, London School of Economics and Political Science, London WC2A 2AE, UK; ^5^ London Mathematical Laboratory, 8 Margravine Gardens, London W6 8RH, UK

**Keywords:** agent-based models, dynamical processes, computational social science

## Abstract

Participants in socio-economic systems are often ranked based on their performance. Rankings conveniently reduce the complexity of such systems to ordered lists. Yet, it has been shown in many contexts that those who reach the top are not necessarily the most talented, as chance plays a role in shaping rankings. Nevertheless, the role played by chance in determining success, i.e. serendipity, is underestimated, and top performers are often imitated by others under the assumption that adopting their strategies will lead to equivalent results. We investigate the tradeoff between imitation and serendipity in an agent-based model. Agents in the model receive payoffs based on their actions and may switch to different actions by either imitating others or through random selection. When imitation prevails, most agents coordinate on a single action, leading to non-meritocratic outcomes, as a minority of them accumulate the majority of payoffs. Yet, such agents are not necessarily the most skilled ones. When serendipity dominates, instead, we observe more egalitarian outcomes. The two regimes are separated by a sharp transition, which we characterize analytically in a simplified setting. We discuss the implications of our findings in a variety of contexts, ranging from academic research to business.

## 1. Introduction

Rankings of individuals, companies and institutions based on their performance have become ubiquitous, reducing complex systems to ordered lists that reflect the ability of their participants to perform precise functions [1]. Rankings are used in a variety of domains, ranging from natural to social, and from economic to infrastructural ones [2], with the aim of a better allocation of resources, funds and rewards [3]. The emphasis on rankings has spurred considerable interest in their dynamics over time [4], leading to the birth of a novel research field named ‘ranking of rankings’, which investigates the goodness of a ranking’s evaluation criteria [5].

One of the main functions of rankings is to help identify the strategies of top performers, which are often recognized as the best practices that others should adopt. In fact, individuals, organizations and institutions often assume that they will be equally successful if they imitate top performers’ strategies. However, there is considerable evidence that such an approach can sometimes backfire and result in a weak association between skills and measured performance [6], and that the agents can be more successful when they develop their own strategies [7] or pursue risky ones [6,8] rather than imitate those of others.

Imitation in ranking dynamics is often grounded in social influence, which often drives individuals’ decision-making and shapes the collective wisdom of the crowd [9–12]. A prime example of these effects was illustrated in a much-celebrated experiment in an artificial music market, which resulted in very low correlations between a song’s success when social interactions between market participants were switched on/off [13].

The role played by chance in successful paths is often underestimated. For instance, in [14,15] it was shown that in a synthetic society of agents, luck prevails over talent in favouring an agent’s rise to the top. At the same time, chance rarely compensates over time for self-reinforcing mechanisms usually referred to as the ‘rich-get-richer’ effect or the Matthew effect [16,17], according to which success breeds more success, often to the point that a (sometimes random) early competitive advantage can lead to long-lasting consequences [18]. The interplay between chance and those effects has been extensively investigated in the literature. Recently, an agent-based models has shown that when some degree of skill is necessary to be successful in life, it is rare for the most talented individuals to reach the highest peaks of success, while averagely talented but sensibly luckier individuals reach the top of rankings [14].

In this article, we explore the consequences of imitation versus chance in an artificial society whose agents are ranked based on a notion of performance. After characterizing the agents’ intrinsic skills, we analyse the payoff gained by the society under different scenarios, and show which of those leads to more/less meritocratic outcomes. Our results capture quite nicely some of the aforementioned phenomena. When imitation prevails, the top performers are not the most skilled ones and, at the same time, the most skilled agents are not the ones who accumulate the highest payoffs. On the contrary, when chance is the dominant mechanism, society becomes more meritocratic. In the latter scenario, we can speak of serendipity, namely positive developments of events that occur in an unplanned manner [19].

## 2. Results

### 2.1. Model implementation

**Figure 1.**
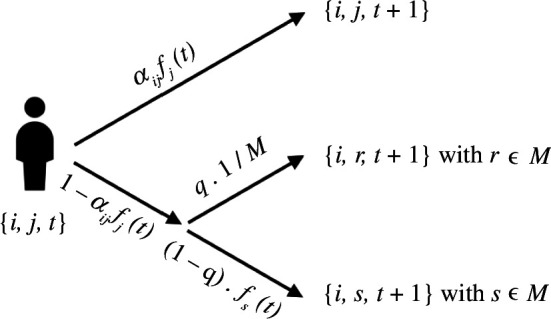
Representation of the model dynamics for a generic agent i who is in state j at time t. All the possible transition outcomes are reported with their probability. The parameter $\alpha_{ij}$ represents the skill of agent i in field j, $f_{j}(t)$ is the fraction of agents playing action j at time t, M is the number of possible actions and the parameter q accounts for randomness in the switching.

We consider an agent-based model with discrete-time dynamics, in which agents have to decide at each time step whether to persist with their current action or switch to a different one. It is conceived as a memory-less process since each move is independent of the previous ones. A schematic representation of the dynamics is shown in figure 1. In more detail, there are N agents, labelled with indices i∈N={1,..., N}, who are empowered to take M actions, labelled with indices j∈M={1,..., M}. In the following, we will write j(i,t) to denote the fact that agent i plays action j at time t. In order to keep our notation as light as possible, we will simply indicate j(i,t) as j whenever possible. Each action j is associated with a societal impact $\pi_{j}\in [0,1]$. Actions with high societal impact ($\pi_{j}\to 1$) have beneficial effects on society as a whole, whereas actions with low societal impact ($\pi_{j}\to 0$) do not. For instance, in the context of academic research, we may think of an action characterized by a high $\pi_{j}$ as a research field such as, e.g. cancer research, whereas an action with low $\pi_{j}$ would correspond to a niche field.

As mentioned above, agents in the model may switch to a different action at any given time step. The switching mechanism is not trivial and depends on a few factors. Among these, we find an agent’s skills, defined through a random matrix whose elements are denoted with $\alpha_{ij}\in [0,1]$. A high value of $\alpha_{ij}$ indicates that agent i is skilled at action j, a low value indicates the opposite. Note that both $\alpha_{ij}$ and $\pi_{j}$ are randomly extracted from a uniform distribution in [0,1] and are assigned at the beginning of each simulation. Another relevant quantity is the appeal of each action, represented by the fraction of agents $f_{j}(t)$ who are performing action j at time t. We identify an action’s appeal with its popularity to encapsulate some of the mechanisms discussed in §1, namely the effects of imitation and social influence on individual choices.

The model’s dynamics work as follows. At time t=0 agents are allocated to the available actions with probability $\pi_{j}$, i.e. proportionally to the actions’ societal impact. At each time step t=0,...,T, each agent receives a payoff $\alpha_{ij}\;f_{j}(t)$. This assumption reflects the fact that agents will receive higher payoffs when they play actions that they are good at and/or that are popular, regardless of their societal impact. In fact, a large $f_{j}(t)$ may compensate for a relatively low $\alpha_{ij}$. Let us note that the payoff lies in [0,1].

At $t^{\prime }=t+1$ (with t=0,...,T−1) agents persevere with the action of time t with probability equal to the previous time step’s payoff, i.e. $\alpha_{ij}\;f_{j}(t)$, reflecting the incentive to stick with actions that are rewarding. In the electronic supplementary material, we implement a slightly different dynamics, for which agents persevere in their current action with probability $\alpha_{ij}\;f_{j}(t)\;\pi_{j}$, i.e. by taking into account an action’s societal impact as well.

In the baseline scenario, an agent will choose to switch actions with probability $1-\alpha_{ij}\;f_{j}(t)$. In this case, an additional parameter q∈[0,1] is introduced to interpolate between imitation and chance. Namely, with probability q agents pick a new action at random. With complementary probability 1−q, they pick their new action with probability $f_{j}(t)$, i.e. proportionally to its current popularity.

In this respect, q quantifies to what extent agents pay attention to the rankings induced by the accumulation of payoffs (see next section) and choose to imitate the actions of their peers [7]: when q→0, imitation becomes the dominant mechanism, while if q→1 agents randomly explore the space of available actions. Let us remark here that high values of q are amenable to different interpretations. In fact, q→1 may be interpreted either as genuine serendipity or as an intrinsic propensity of the agents to explore different options. Being deliberately stylized, our model cannot distinguish between such interpretations. For the sake of simplicity, throughout the rest of the article, we shall mostly refer to serendipity when q→1. However, we will briefly explore a different model specification aimed at partially highlighting the difference between serendipity and exploration.

We implement simulations of our model for several values of q to achieve a comprehensive understanding of the different scenarios. For each parameter set, we run S=1000 simulations to ensure robust statistical analysis, and we let each simulation run for T=500 time steps, for which a steady or absorbing state (depending on the value) is reached. We checked that outcomes of the simulations do not vary when we keep the ratio N/M fixed, so in the following we will use N=250 agents and M=100 actions. The rationale behind the choice of N>M is that there are likely more agents than possible actions to play in the most realistic settings. Anyway, the qualitative outcomes of the model for N≶M are the same unless explicitly mentioned. Error bars in the figures represent sample standard deviations.

### 2.2. Success and social disparity

To investigate the outcome(s) of different scenarios, it is necessary to introduce some observables. First, we define the cumulative payoff that agent i gains with their actions over time, from t=0 to t=T, as


(2.1) $$P_{i}\left(T\right)=\sum_{t=0}^{T}\alpha_{ij}f_{j}\left(t\right)\text{.}$$

We remind the reader that in the above and the following equations j=j(i,t), i.e. the action played by agent i at time t. The overall societal payoff collectively generated by all agents is thus given by


(2.2) $$P\left(T\right)=\sum_{i=1}^{N}\sum_{t=0}^{T}\alpha_{ij}f_{j}\left(t\right)\text{.}$$

**Figure 2.**
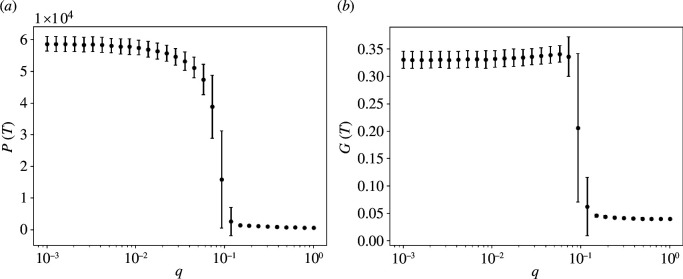
(*a*) Cumulative societal payoff P(T) for several values of the parameter q. (*b*) Gini coefficient G(T) for the same values of q. Simulations have been implemented with N=250 agents, M=100 actions and T=500 time steps. Results are an average over a sample of size S=1000.

We combine the analysis of P(T) with the study of the Gini coefficient, a measure of inequality in a society, which is defined as


(2.3) $$G\left(T\right)=\frac{1}{N\;P\left(T\right)}\sum_{i<k}|P_{i}\left(T\right)-P_{k}\left(T\right)|\text{,}$$

leading to G(T)=0 for perfect equality and G(T)→1 when cumulative payoffs are concentrated in the hands of very few agents. The results of these investigations are shown in figure 2. When imitation is the prevailing mechanism, i.e. q→0, the overall payoff P(T) reaches its maximum, though this happens at a cost. In fact, the Gini coefficient G(T) shows that this corresponds to an unequal society, in which the majority of the payoff is generated by a minority of the agents. On the contrary, when randomness dominates (for q→1), we observe a lower societal payoff distributed more equally. Between these two limiting conditions, a sharp transition happens for a certain value $q^{*}$, for which we provide some intuition in a simplified setting in the following section. Note that error bars are particularly large at the phase transition, in line with behaviours observed for phase transitions in condensed matter physics.

It is fair to wonder which of the two above societies represents a better outcome: a ‘richer’ but unequal one or a ‘poorer’ but more egalitarian one? Intuitively, the former would be preferable when higher individual payoffs reflect an agent’s superior skills, i.e. when outcomes are meritocratic. To this aim, we compute the Kendall rank correlation coefficient τ— a measure of similarity between two rankings [20]—between the agents’ cumulative payoff $P_{i}(T)$ and intrinsic fitness. We define the latter as the average skill of an agent across all possible actions:


(2.4) $$\phi_{i}^{avg}=\frac{1}{M}\sum_{j=1}^{M}\alpha_{ij}.$$

**Figure 3.**
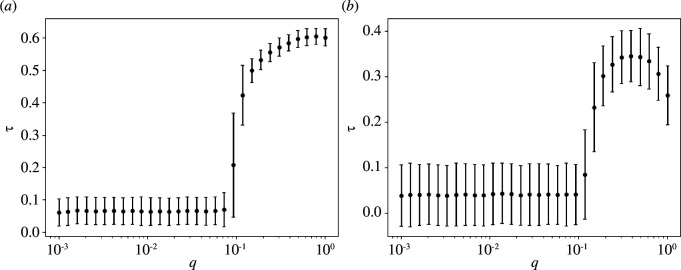
Mean Kendall rank correlation coefficient τ between an agent’s cumulative payoff and their average skill. (*a*) N=250, M=100. (*b*) N=100, M=250. Other simulation parameters are the same as for figure 2.

As shown in figure 3, for $q<q^{*}$, there is no correlation between the agents’ payoffs and fitness. As q increases, we find again a sharp transition at $q=q^{*}$ and, for $q>q^{*}$, some correlation between payoffs and fitness (i.e. meritocracy) is restored. This behaviour changes slightly depending on the ratio N/M. When M>N, τ behaves non-monotonically; when N>M, instead, it increases monotonically. In line with [14], the above results further confirm that the most successful agents (i.e. those who accumulate the highest payoff) in general are not the most skilled ones. Intuitively, this occurs because—when imitation prevails—during the model’s early time steps a few lucky agents ‘stumble’ upon actions that they are good at, resulting in high payoffs. This, in turn, triggers the imitation of other agents, who flock towards the same actions, resulting in a feedback loop that effectively prevents most agents from discovering actions that would be more profitable for them.

Combining the different elements, these analyses show that imitation could be the winning strategy only for a limited number of agents and that the most skilled ones would not be the most successful. On the contrary, when chance dominates, outcomes are serendipitous and society becomes more egalitarian. It should be noted that these results qualitatively do not change even when the agents are given the opportunity to improve their skills when playing actions repeatedly over time (see below the section devoted to time-varying skills).

### 2.3. Condensation and resource allocation

This subsection is dedicated to the investigation of the distribution of the actions played by agents. In particular, we focus on the participation ratio, a measure of concentration borrowed from condensed matter and quantum physics, defined as


(2.5) $$PR(t)=\frac{1}{M}{\left[\sum_{j}{\left({\tilde{f}}_{j}(t)\right)}^{4}\right]}^{-1}\;;\;\;\;\;{\tilde{f}}_{j}(t)=\frac{f_{j}(t)}{\sum_{i}f_{i}^{2}(t)}$$

with j∈M. In the above expression, ${\tilde{f}}_{j}(t)$ is the normalized (in $L^{2}$ norm) counterpart of the quantity $f_{j}(t)$. In a nutshell, the participation ratio effectively counts the fraction of entries that are significantly different from zero in a list. Let us consider two extreme examples. When all agents play the same action, the list ${\tilde{f}}_{j}(t)$ is maximally concentrated, i.e. ${\overset{˜}{f}}_{\ell }\left(t\right)=1$ for some action ℓ and ${\tilde{f}}_{j}(t)=0$ for each other action j≠ℓ, resulting in $PR(t)=M^{-1}$. When, on the contrary, each action is played by an equal number of agents, i.e. ${\tilde{f}}_{j}(t)=M^{-1/2}$ for each action j, one has PR(t)=1.

**Figure 4.**
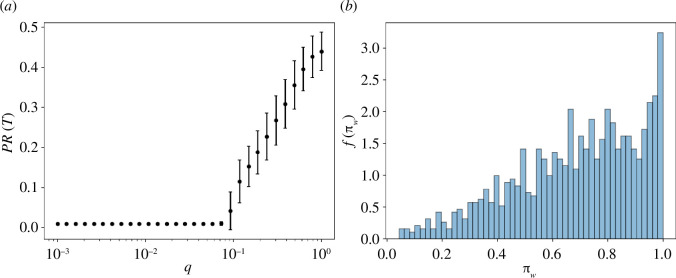
(*a*) Participation ratio PR(T). (*b*) Normalized distribution of the payoffs of the most played actions at time T for q=0. All parameters are the same as for figure 2.

We focus our attention on PR(T). As shown in figure 4, for $q<q^{*}$ all agents condense on a single action s, as reflected by the fact that the participation ratio becomes equal to $M^{-1}$. The action s resembles an absorbing state, as agents can temporarily leave it through the switching mechanism (when q>0), but are bound to return to it through the very same mechanism. After the transition at $q=q^{*}$, other actions start to be populated, as evidenced by the fact that the participation ratio increases monotonically and becomes significantly different from zero. Yet, it always remains well below one, signalling that a substantial fraction of actions do not get played by any agent. We further elaborate on these points in the section devoted to analytical considerations below.

Condensation on a single action is a positive outcome when such action is beneficial to society. For that reason, we study the distribution across simulations of the societal payoffs for the most played action, which we refer to as the ‘winning’ one, when q=0. As we can see from figure 4*b*, in the majority of simulations the winning activity is associated with an above-average societal payoff ($\pi_{w}>1/2$). Yet, in a sizeable minority of cases (approximately 20% of simulations) it is not unlikely for the agents to coordinate on an action with a below average societal payoff as a consequence of imitation. It is remarkable that the same scenario occurs even when the societal payoff $\pi_{j}$ is explicitly accounted for in the switching mechanism, namely agents keep playing the same action with probability proportional to $\alpha_{ij}\;f_{j}(t)\;\pi_{j}$, as shown in the electronic supplementary material.

Finally, we aim to address the following question: are the agents playing actions that are beneficial to society good at them? To this end, we introduce a simple measure of societal benefit


(2.6) $$B\left(t\right)=\sum_{i=1}^{N}\;\alpha_{ij\left(i,t\right)}\;\pi_{j\left(i,t\right)}$$

whose cumulative value over time is given by $B(T)=\sum_{t=0}^{T}B(t)$. Let us remind the reader that in the above expression j(i,t) denotes that agent i undertakes action j at time step t. The above quantity, therefore, depends both on time and on the list of actions being played by the agent population at that time. We omit the latter dependence to keep the notation as light as possible. Higher values of B(T) indicate that the model’s dynamics naturally incentivize agents to play actions that generate societal benefits when they are good at them.

**Figure 5.**
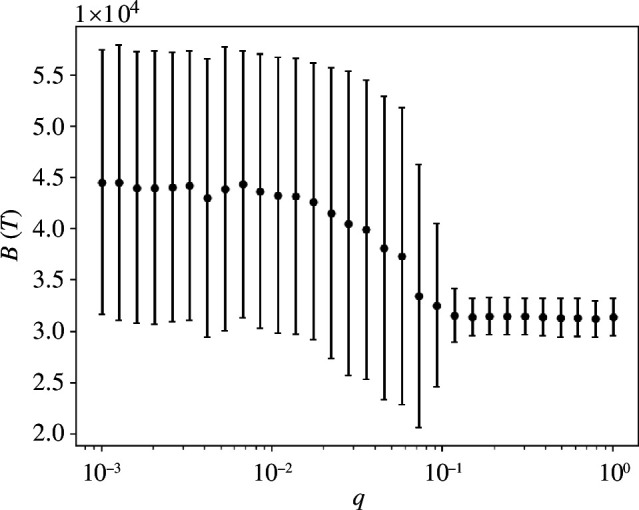
The overall benefit B(T) gained by the community owing to agents’ abilities in socially beneficial actions. Simulation parameters are the same as for the previous analyses.

As shown in figure 5, on average B(T) is higher for $q<q^{*}$, yet its standard deviation is particularly large in this regime. After the transition, $q>q^{*}$ societal benefit decreases, together with its standard deviation.

### 2.4. Different model specifications

In this section, we briefly explore different specifications of our model aimed at addressing some of its possible limitations. First, we consider a version of the model in which the agent population is split into a sub-group of agents characterized by different values of q. We do this to account for the fact that what we refer to as serendipity in our model may also be interpreted as an agent’s propensity to explore different options in the action space, in line with game-theoretic literature in economics [21,22]. In this respect, when the two sub-groups are characterized by very low and very high values of q, respectively, we can study the model’s outcomes produced by the interaction between ‘explorer’ (high q) and ‘imitator’ (low q) agents. Obviously, when one of the two sub-groups becomes exceedingly more numerous than the other, the model produces results which are identical to those obtained with a homogeneous population characterized by a low/high value of q. Therefore, we study this version of the model only in settings where neither of the two sub-group dominates. We do so by keeping q fixed to a low value in one sub-group and varying its value in the other one. The results are reported in the electronic supplementary material, and are qualitatively very similar to the ones obtained with a homogeneous population. Namely, we again observe a transition when q in the other sub-group exceeds a critical value. Once again, this critical value separates regimes characterized, respectively, by high/low inequality and low/high correlation between an agent’s fitness and their accumulated payoff.

We also explore the model’s behaviour when the agents become better at playing a given action when given the opportunity to do so repeatedly over time. So far, we have considered an agent’s skill set $\alpha_{ij}$ to remain constant over time. Here, we instead allow the $\alpha_{ij}$’s to evolve over time with the following simple rule. Whenever an agent i plays a given action j at time t, they receive a reward that depends on their skill at playing such action $\alpha_{ij}(t)$. Should they continue to play such action, their skill will *increase* to a new value $\alpha_{ij}(t+1)$, drawn at random from the uniform distribution on the interval $\left[\alpha_{ij}(t),1\right]$.

**Figure 6.**
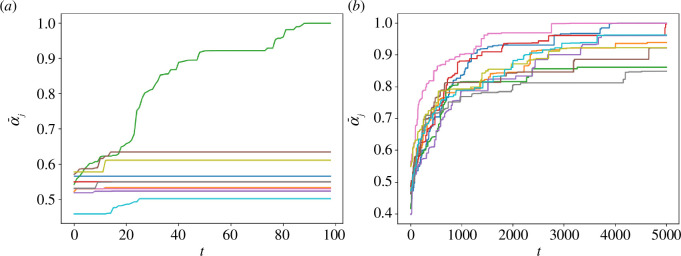
Temporal evolution of the average agent skill ${\bar{\alpha }}_{j}(t)=\sum_{i=1}^{N}\alpha_{ij}(t)/N$ in a model with time-varying skills, N=25, M=10. (*a*) q=0.1. (*b*) q=0.8.

The typical dynamics that result from the above rule are shown in figure 6, where we represent the temporal evolution of ${\bar{\alpha }}_{j}(t)=\sum_{i=1}^{N}\alpha_{ij}(t)/N$, i.e. the average agent skill at playing action j. Figure 6*a* reports the results in an imitation-driven setting (q=0.1), whereas figure 6*b* shows results obtained in a serendipity-driven setting (q=0.8). As it can be seen, when imitation prevails the agents eventually all become excellent at one action (the one played by the entire population in the long run), and basically do not improve their other skills. Conversely, when imitation is low, all agents eventually become good at playing all strategies, with ${\bar{\alpha }}_{j}(t)\to 1,\forall \;j$, for sufficiently long times.

In the electronic supplementary material, we report results for the payoff–fitness correlation and societal benefit obtained with this version of the model, illustrating that they do not qualitatively differ from those obtained with constant skills.

### 2.5. Analytical considerations

In order to gain some analytical insights on the model’s behaviour, we consider a simplified version with M=2 activities and $\alpha_{ij}=\alpha$ (∀i, j=1,2). We define $n_{j}(t)$ as the number of agents who play action j at time t, so that $n_{1}(t)+n_{2}(t)=N$. In addition, we replace the model’s probabilistic dynamics with deterministic transition rates. According to the dynamics shown in figure 1, we study the flux of agents who enter and exit from activity 1 at time $t^{\prime }=t+1$. Focusing on agents who play action 1 at time t, at $t^{\prime }=t+1$ there are

– $\alpha \;f_{1}(t)\;n_{1}(t)$ agents who at time t are taking action 1 and choose to keep playing it;
– $\left(1-\alpha \;f_{1}(t))\;n_{1}(t\right)$ agents who consider leaving activity 1. Out of this group, $q\;(1-\alpha \;f_{1}(t))\;n_{1}(t)/2$ agents keep action 1 as a consequence of random selection and $\left(1-q)\;f_{1}(t)\;(1-\alpha \;f_{1}(t))\;n_{1}(t\right)$ agents pick action 1 because of its popularity.

Correspondingly, examining agents who are in action 2 at time t, we have a total of $\left(1-\alpha \;f_{2}(t))\;n_{2}(t\right)$ agents who consider leaving it. Out of these, $q\;(1-\alpha \;f_{2}(t))\;n_{2}(t)/2$ agents end up in action 1 through random selection and $\left(1-q)\;f_{1}(t)\;(1-\alpha \;f_{2}(t))\;n_{2}(t\right)$ agents end up in it because of its popularity.

Considering the relations $n_{2}(t)=N-n_{1}(t)$ and $f_{2}(t)=1-f_{1}(t)$, we combine the previous contributions and write:


(2.7) $$n_{1}\left(t+1\right)=n_{1}\left(t\right)\;\left[\alpha \;f_{1}\left(t\right)+\left(1-\alpha \;f_{1}\left(t\right)\right)\left(\frac{q}{2}+\left(1-q\right)\;f_{1}\left(t\right)\right)\right]+\left(N-n_{1}\left(t\right)\right)\left(1-\alpha \left(1-f_{1}\left(t\right)\right)\right)\left(\frac{q}{2}+\left(1-q\right)f_{1}\left(t\right)\right).$$

We now convert the previous relation into an equation for the increment $n_{1}(t+1)-n_{1}(t)$. After a few simplifications, dividing by N and taking the continuous limit ultimately delivers an equation for the time derivative $f_{1}^{\prime }(t)$:


(2.8) $$f_{1}^{\prime }\left(t\right)=\left(2\;f_{1}\left(t\right)-1\right)\left[\alpha f_{1}\left(t\right)\left(1-f_{1}\left(t\right)\right)\left(1-q\right)+\frac{q}{2}\left(\alpha -1\right)\right].$$

**Figure 7.**
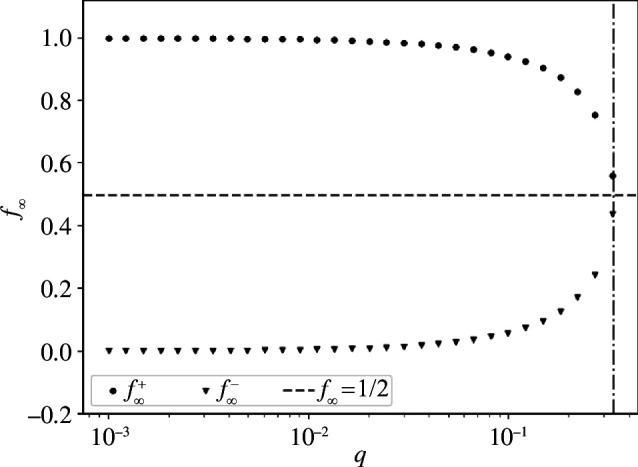
Fraction of agents $f_{\infty }$ as a steady-state solution in a simplified scenario with two available actions. The horizontal line marks the trivial solution $f_{\infty }=1/2$, while the vertical one underlines the value $q=q^{*}$ for which a sharp transition occurs.

After writing the corresponding equation for $f_{2}^{\prime }(t)$, it can be shown that $f_{1}^{\prime }(t)+f_{2}^{\prime }(t)=0$, as it should be. Therefore, the system is effectively described by just one differential equation. We choose the one in equation (2.8), we remove the index 1 from f and set $f^{\prime }(t)=0$ to find steady-state solutions. This is a cubic equation, yielding three solutions $f_{\infty }=\lim_{t\to \infty }f(t)$ which can be expressed as functions of q and α. One solution is trivial and equal to the constant 1/2, reflecting the fact that an initial condition in which $f_{1}(0)=f_{2}(0)=1/2$ remains unchanged. The two non-trivial solutions read


(2.9) $$f_{\infty }^{\pm }=\frac{1}{2}\;\pm \frac{1}{2}\sqrt{\frac{q\left(\alpha -2\right)+\alpha }{\alpha \left(1-q\right)}},$$

with $f_{\infty }^{+}+f_{\infty }^{-}=1$, capturing the fact that each solution describes the popularity of one of the two fields in the stationary state. Notably, from the above expression we can see that $f_{\infty }^{\pm }$ are real numbers only for q≤α/(2−α), which we can then identify with the threshold $q^{*}$ in this simplified two-state setting. This, in fact, is the point where the constant solution $f_{\infty }^{\pm }=1/2$ becomes the stable one. In figure 7, we show the three steady-state solutions as functions of q for α=1/2, resulting in $q^{*}=1/3$. Notably, this holds despite the fact that such critical value is obtained under the assumption of a constant value for α, which in this case is assumed to be equal to its average value, i.e. α=〈α〉=1/2. All in all, this highly stylized version of our model is sufficient to illustrate that a threshold separates a regime where actions are equally played from one where one action dominates, eventually resulting in condensation when q→0.

## 3. Discussion

Individuals and organizations are encouraged to imitate the behaviour of top performers in rankings. However, there is mounting evidence that this strategy is not the best one [6,7,14]. In this article, we further support this assertion by considering an agent-based model to investigate the role of imitation and serendipity in social dynamics. In particular, we study the cumulative payoff that agents gain by choosing actions within a set based on these two mechanisms. We find that society reaches a higher payoff when imitation prevails, but at the cost of higher inequality. Moreover, in this scenario, we find no correlation between an agent’s accumulated payoff and their fitness. This is evident in the fact that the most skilled agents are not necessarily the most successful ones, clearly indicating that society is not meritocratic.

We also examine how agents distribute themselves among the available actions. Our conclusion is that, when imitation is the predominant mechanism diversity is significantly reduced, as all agents tend to concentrate on one single action, which may not even be associated with meaningful societal benefits. On the contrary, when decisions are left to chance, outcomes become more serendipitous. In fact, the society becomes more egalitarian, with a higher correlation between payoffs and skills, and a significant portion of available actions are played at any given time. These results are reminiscent of some of the findings published in [14,15]. However, let us remark that the driving mechanisms underpinning the two models are different, as we consider interactions between agents through imitation.

The two extremal conditions are separated by a sharp transition. Notably, sharp transitions have been observed in other social systems where agents tend to conform to one another, e.g. in social climbing phenomena [23], in the formation of political parties with a strong leader [24], in stock markets [25] or even in the sudden emergence of traffic jams [26]. This transition can be related to mechanisms which are similar to the ones responsible for phase transitions in physical systems [27].

Our model is rather stylized, but at the same time, extremely general, as we deliberately avoid to specify the details of the social system it describes. As such, its agents and their interactions capture a wide variety of situations. For instance, when the COVID-19 pandemic broke out it immediately became—through imitation—the main theme of research in most STEM fields, and in just a few months an incredible volume of papers on this subject were published [28]. Notably, the rush led to several documented errors in methodology and conclusions [29]. In fact, in a ‘publish or perish’ [30] environment incentives—both at the level of authors and publishers—often favour condensation-like phenomena, i.e. the crowding towards certain research topics.

Switching to an entirely different domain, agents in our model may be interpreted as entrepreneurs starting a new business, needing to decide whether they should pursue the latest trend or something novel. The literature on the recent startup bubble shows that imitation-based entrepreneurial strategies lead to mixed results [31], which is in line with the outcomes produced by our model.

A possible extension of our model could incorporate time-varying societal payoffs, i.e. allowing the $\pi_{j}$ parameters to be functions of time. This could capture the fact that the importance of certain actions could change owing to exogenous events impacting society. For instance, going back to the aforementioned example, the sudden emergence of the COVID-19 pandemic obviously drove a massive change in the importance of the research topics related to it. In this respect, our model captures scenarios in which the agents’ decision-making evolves over time scales that are much shorter than those that characterize changes in the societal payoffs of actions.

## Data Availability

Data and relevant code for this research work are stored in GitHub [32] and Zenodo 10.5281/zenodo.11920282 [33] and 10.5281/zenodo.12706176 [34]. Electronic supplementary material is available online [35].

## References

[1] Iñiguez G, Pineda C, Gershenson C, Barabási AL. 2022 Dynamics of ranking. Nat. Commun. **13**, 1646. (10.1038/s41467-022-29256-x) 35347126 PMC8960905

[2] Érdi P. 2019 Ranking: the unwritten rules of the social game we all play. Oxford, UK: Oxford University Press. (10.1093/oso/9780190935467.001.0001)

[3] Blumm N, Ghoshal G, Forró Z, Schich M, Bianconi G, Bouchaud JP, Barabási AL. 2012 Dynamics of ranking processes in complex systems. Phys. Rev. Lett. **109**, 128701. (10.1103/PhysRevLett.109.128701) 23005999

[4] Sun Y, Caccioli F, Livan G. 2023 Ranking mobility and impact inequality in early academic careers. Proc. Natl Acad. Sci. USA **120**, e2305196120. (10.1073/pnas.2305196120) 37579179 PMC10450398

[5] Stolz I, Hendel DD, Horn AS. 2010 Ranking of rankings: benchmarking twenty-five higher education ranking systems in Europe. High. Educ. **60**, 507–528. (10.1007/s10734-010-9312-z)

[6] Denrell J, Liu C. 2012 Top performers are not the most impressive when extreme performance indicates unreliability. Proc. Natl Acad. Sci. USA **109**, 9331–9336. (10.1073/pnas.1116048109) 22645350 PMC3386112

[7] Livan G. 2019 Don’t follow the leader: how ranking performance reduces meritocracy. R. Soc. Open Sci. **6**, 191255. (10.1098/rsos.191255) 31827860 PMC6894586

[8] Guedj O, Bouchaud J-P. 2005 Experts’ earning forecasts: bias, herding and gossamer information. Int. J. Theor. Appl. Finan. **8**, 933–946. (10.1142/S0219024905003281)

[9] Lorenz J, Rauhut H, Schweitzer F, Helbing D. 2011 How social Influence can undermine the wisdom of crowd effect. Proc. Natl Acad. Sci. USA **108**, 9020–9025. (10.1073/pnas.1008636108) 21576485 PMC3107299

[10] Mannes AE. 2009 Are we wise about the wisdom of crowds? The use of group judgments in belief revision. Manage. Sci. **55**, 1267–1279. (10.1287/mnsc.1090.1031)

[11] Bond R, Smith PB. 1996 Culture and conformity: a meta-analysis of studies using Asch’s (1952b, 1956) line judgment task. Psychol. Bull. **119**, 111–137. (10.1037/0033-2909.119.1.111)

[12] Cialdini RB, Goldstein NJ. 2004 Social Influence: compliance and conformity. Annu. Rev. Psychol. **55**, 591–621. (10.1146/annurev.psych.55.090902.142015) 14744228

[13] Salganik MJ, Dodds PS, Watts DJ. 2006 Experimental study of inequality and unpredictability in an artificial cultural market. Science **311**, 854–856. (10.1126/science.1121066) 16469928

[14] Pluchino A, Biondo AE, Rapisarda A. 2018 Talent versus luck: the role of randomness in success and failure. Advs. Complex Syst. **21**, 1850014. (10.1142/S0219525918500145)

[15] Challet D, Pluchino A, Biondo AE, Rapisarda A. 2020 The origins of extreme wealth inequality in the talent versus luck model. Advs. Complex Syst. **23**, 2050004. (10.1142/S0219525920500046)

[16] Merton RK. 1968 The Matthew effect in science. Science **159**, 56–63. (10.1126/science.159.3810.56) 5634379

[17] Petersen AM, Fortunato S, Pan RK, Kaski K, Penner O, Rungi A, Riccaboni M, Stanley HE, Pammolli F. 2014 Reputation and impact in academic careers. Proc. Natl Acad. Sci. USA **111**, 15316–15321. (10.1073/pnas.1323111111) 25288774 PMC4217436

[18] Li W, Aste T, Caccioli F, Livan G. 2019 Early coauthorship with top scientists predicts success in academic careers. Nat. Commun. **10**, 5170. (10.1038/s41467-019-13130-4) 31729362 PMC6858367

[19] Yaqub O. 2018 Serendipity: towards a taxonomy and a theory. Res. Policy **47**, 169–179. (10.1016/j.respol.2017.10.007)

[20] Kendall MG. 1938 A new measure of rank correlation. Biometrika **30**, 81–93. (10.1093/biomet/30.1-2.81)

[21] Young HP. 1993 The evolution of conventions. Econometrica **61**, 57. (10.2307/2951778)

[22] Young HP. 2020 Individual strategy and social structure: an evolutionary theory of institutions. Princeton, NJ: Princeton University Press. (10.2307/j.ctv10h9d35)

[23] Bardoscia M, De Luca G, Livan G, Marsili M, Tessone CJ. 2013 The social climbing game. J. Stat. Phys. **151**, 440–457. (10.1007/s10955-013-0693-0)

[24] Hołyst JA. 2023 Why does history surprise us? J. Comput. Sci. **73**, 102137. (10.1016/j.jocs.2023.102137)

[25] Kirman A. 1993 Ants, rationality, and recruitment. Q. J. Econ. **108**, 137–156. (10.2307/2118498)

[26] Kerner BS, Rehborn H. 1997 Experimental properties of phase transitions in traffic flow. Phys. Rev. Lett. **79**, 4030–4033. (10.1103/PhysRevLett.79.4030)

[27] Levy M. 2005 Social phase transitions. J. Econ. Behav. Organ. **57**, 71–87. (10.1016/j.jebo.2003.11.013)

[28] Teixeira da Silva JA, Tsigaris P, Erfanmanesh M. 2021 Publishing volumes in major databases related to COVID-19. Scientometrics **126**, 831–842. (10.1007/s11192-020-03675-3) 32904414 PMC7454548

[29] Capodici A, Salussolia A, Sanmarchi F, Gori D, Golinelli D. 2023 Biased, wrong and counterfeited evidences published during the COVID-19 pandemic, a systematic review of retracted COVID-19 papers. Qual. Quant. **57**, 4881–4913. (10.1007/s11135-022-01587-3) PMC9707851 36466994

[30] De Rond M, Miller AN. 2005 Publish or perish: bane or boon of academic life? J. Manag. Inq. **14**, 321–329. (10.1177/1056492605276850)

[31] Tsolakidis P, Mylonas N, Petridou E. 2020 The impact of imitation strategies, managerial and entrepreneurial skills on startups’ entrepreneurial innovation. Economies **8**, 81. (10.3390/economies8040081)

[32] Livan G. 2024 Imitation_Vs_Serendipity. GitHub. See https://github.com/giacomolivan/imitation_vs_serendipity.

[33] Livan G. 2024 Code for the paper: Imitation vs serendipity in ranking dynamics. Zenodo. (10.5281/zenodo.11920282) PMC11265917 39050725

[34] Livan G. 2024 Giacomolivan/imitation_vs_serendipity: v10. Zenodo. (10.5281/zenodo.12706176)

[35] De Domenico F, Caccioli F, Livan G, Montagna G, Nicrosini, O. 2024 Data from: Imitation versus serendipity in ranking dynamics. Figshare. (10.6084/m9.figshare.c.7358205) PMC11265917 39050725

